# Hyperbaric oxygen therapy alleviates intestinal dysfunction following traumatic brain injury via m^6^A regulation

**DOI:** 10.7150/ijms.97682

**Published:** 2024-08-19

**Authors:** Xuelai Yu, Wei Zhao, Yunyun Liu, Jingchuan Lv, Xiang Zhong, Peizan Huang

**Affiliations:** 1 Department of Hyperbaric Oxygen, The Fourth Affiliated Hospital of Nanjing Medical University, 210031 Nanjing, China.; 2 Department of Neurosurgery, The Fourth Affiliated Hospital of Nanjing Medical University, 210031 Nanjing, China.; 3 Department of Pathology, The Fourth Affiliated Hospital of Nanjing Medical University, 210031 Nanjing, China.; 4 Department of Intensive Care Unit, Nanjing Tongren Hospital, School of Medicine, Southeast University, 211102 Nanjing, China.; 5 College of Animal Science and Technology, Nanjing Agricultural University, 210095 Nanjing, China.

**Keywords:** hyperbaric oxygen therapy, traumatic brain injury, intestinal dysfunction, m^6^A

## Abstract

Hyperbaric oxygen (HBO) therapy can attenuate neurological impairment after traumatic brain injury (TBI) and alleviate intestinal dysfunction. However, the role and mechanism of HBO therapy in intestinal dysfunction following TBI remain unclear. Herein, by establishing a mouse model of controlled cortical impact (CCI), we found that HBO therapy reduced histopathological lesions and decreased the levels of inflammatory and oedema proteins in the intestinal tissues of mice 10 days after TBI. We also showed that HBO therapy improved microbiome abundance and probiotic (particularly *g_Bifidobacterium*) colonisation in mice post-CCI. Then, we identified that the m^6^A level imcreased notably in injured cortical tissue of CCI+HBO group compared with the CCI group following CCI. Thus, our results suggested that HBO therapy could alleviate TBI-induced intestinal dysfunction and m^6^A might participate in this regulation process, which provides new insights for exploring the specific mechanism and targets of HBO in the treatment of intestinal dysfunction after TBI, thereby improving the therapeutic effect of HBO.

## Introduction

Traumatic brain injury (TBI) is a major public health concern; severe brain injury develops rapidly and is the leading cause of death from trauma^1^. Approximately 69 million TBIs occur worldwide annually 2, 3. TBI can affect downstream communication from the brain to the gut in the brain-gut axis (BGA), including afferent and efferent signals involving crosstalk between neurones, hormones, and immunity, resulting in disorders of immune regulation, afferent nerve signals, blood-brain barrier, intestinal barrier function, digestion, and absorption^4^. Although the number of TBI-related deaths has decreased markedly, effective therapies to promote the recovery of TBI patients are still lacking. Therefore, there has been a major shift in TBI studies regarding neural recovery^5^.

Several animal and clinical studies have shown that hyperbaric oxygen (HBO) can promote the recovery of neurological function and improve cognitive function and prognosis of TBI^6^-^8^. Other studies have confirmed that HBO therapy can improve intestinal epithelial barrier dysfunction after spinal cord injury in rats^9^. In addition, HBO treatment can ameliorate intestinal microbiota disorders after chronic stress^10^. However, the therapeutic effect of HBO on intestinal dysfunction following TBI remains unclear, and the intervention mechanism of HBO therapy on intestinal dysfunction caused by TBI requires further exploration.

N6-methyladenosine (m^6^A) is one of the most common post-transcriptional mRNA modifications in eukaryotes and is involved in various immune and inflammatory responses^11^. m^6^A plays an important role in both the brain and gut. The m^6^A modification regulates the activation of various nerve conduction pathways and plays a significant role in the development, differentiation, and regeneration of neurones 12,13. Furthermore, m^6^A has a crucial effect on communication between the gut microbiome and the host^14^, ^15^. In our previous study, we confirmed that YTHDF1 (one of m^6^A reading proteins) knockout could reduce TBI-induced BGA dysfunction in mice, wherein we found that YTHDF1 knockout decreased the level of inflammatory proteins in brain and intestinal tissue after TBI^2^. However, whether m^6^A participates in HBO therapy for TBI-induced intestinal dysfunction remains elusive. To confirm the therapeutic effect of HBO on intestinal dysfunction post-TBI, we performed controlled cortical impact (CCI) in C57BL/6J mice; assigned the HBO therapy group to the intervention group; and compared differences in brain defect area and surviving neuron count in brain tissues, the ratio of villus height to crypt depth (V/C), levels of inflammation and oedema proteins in intestinal tissues, and composition of the faecal microbiome. Finally, we identified the differentially expressed levels of m^6^A to determine whether m^6^A is involved in the process of HBO therapy in TBI-induced intestinal dysfunction.

## Materials and Methods

### Animals and grouping

C57BL/6J male mice, aged 8-12 weeks and weighing 25±4 g, were provided by the Animal Centre of Nanjing Agricultural University (Jiangsu, China). The mice were housed in a standardised SPF animal laboratory at a constant temperature (24 °C) and humidity (50%) under a 12 h light-dark cycle and allowed free access to food and water. They were randomly divided into two groups after 1 week of acclimation: (1) CCI group (n=9) and (2) CCI + HBO group (n=9). There were no marked differences between the two groups in terms of general health, locomotor activity, reactivity, or neurological reflexes.

### CCI procedure

Before inducing CCI injury, sodium pentobarbital (65 mg/kg) was injected intraperitoneally to anaesthetise the mice, and the operation was initiated once pedal reflexes were no longer present. The core body temperature was maintained at 37 °C through a heating pad during surgery. The heads of the mice were fixed in a stereotaxic frame, and a 4-mm-diameter craniotomy was performed at 2.0 mm posterior to the bregma and 2.0 mm lateral to the midline over the right hemisphere. A 3.0-mm rounded metal tip attached to the Pin-Point CCI device (Model PCI3000, Hatteras Instruments Inc., Cary, NC, USA) was angled vertically towards the brain surface. A severe injury was performed with a 2.0-mm depth, 3.0 m/s speed, and 180 ms procedural duration^2^ (Supplemental figure 1). The mice were removed from the stereotaxic holder after surgery, and the wound was lightly sutured. Body temperature was maintained in heated cages after the operation, and the mice were returned to their original cages after they were fully awake.

### Hyperbaric oxygen treatment

Mice in the CCI+HBO group began receiving HBO treatment in an experimental hyperbaric chamber 4 h after surgery. The chamber was flushed with 100% O_2_ for 5 min to avoid carbon dioxide accumulation. Compression to 2.5 atmospheres absolute pressure (ATA) was performed for 15 min, followed by maintaining the pressure at 2.5 ATA while inhaling 100% oxygen for 60 min. Subsequently, decompression to normobaric air was conducted for 15 min^16^,^17^. In the whole process, The mice were allowed free access to food and water in the chamber. According to the above treatment regimen, mice received HBO treatment once daily for 10 consecutive days. The mice in the CCI group inhaled 21% oxygen at 1.0 ATA postoperatively.

### Sample collection

One mouse died in the CCI+HBO group and three mice died in the CCI group. They were supplemented and given the same CCI and HBO treatments. Haematoxylin and eosin (HE) staining was performed on three mice in each of the two groups 10 days after CCI. Mice were euthanised via intraperitoneal injection of sodium pentobarbital (65 mg/kg) and perfused transcardially with phosphate-buffered saline, followed by 50 mL of 4% paraformaldehyde. The brains were promptly removed from the mouse body and fixed in 4% paraformaldehyde at 4 °C for 48 h. A vibratome (Leica VT 1000S, Wetzlar, Germany) was used to obtain coronal sections containing the entire hippocampus (-0 mm, -3.5 mm) relative to the bregma. A cryostat (Leica CM 1950) was used to cut serial coronal sections (30 μm thick) for HE staining (*n* = 3 per group). Jejunal tissue was excised, fixed in 4% paraformaldehyde solution, dehydrated using ethanol and xylene, and embedded in paraffin. In addition, 5 mm-thick sections were cut for HE staining. Furthermore, the injured cerebral cortex (respective n=6) and jejunal tissue (respective n=6) were promptly dissected, weighed, frozen in liquid nitrogen, and then stored at -80 °C for enzyme-linked immunosorbent assay (ELISA). Moreover, the faecal samples (respective n=3) were promptly collected, weighed, frozen in liquid nitrogen, and then stored at -80 °C for SMRT sequencing.

### HE

Jejunal and brain sections were rinsed with dH_2_O, stained with haematoxylin for 6 min, and then decolourised in acid alcohol for 1 s. Before immersion in LiCO_3_, sections were rinsed with dH_2_O for 3 s and counterstained with eosin for 15 s. The sections were rinsed with dH_2_O and dehydrated with 95% ethyl alcohol for 2-3 minutes and 100% ethyl alcohol for 2-3 minutes. Subsequently, the sections were cleared with xylene for 2-5 min, mounted with DePeX (Thermo Fisher Scientific Inc., Waltham, MA, USA) in a fume hood, and visualized using an inverted microscope at 100× magnification (Nikon, Tokyo, Japan). Digital images were captured using a SPOT microscope camera (Diagnostic Instruments, Sterling Heights, MI, USA).

### ELISA

The purified proteins were resuspended in carbonate buffer pH 9.6 at a concentration of 5 μg/ml, dispensed into 96-well polystyrene plates at a final volume of 50 μl per well, and fixed overnight at 4 °C. The plates were then blocked with phosphate-buffered saline (PBS) and 5% milk for 1 h at 22-26 °C. Next, the sera dilution was performed in PBS and 1% milk at a concentration of 1/200. Serum samples were incubated for 1 h at 37 °C. After three washes with PBS-Tween 0.05%, the cells were incubated with a secondary antibody, anti-human immunoglobulin G (IgG), coupled with peroxidase (Invitrogen Life Technologies, Frederick, MD, USA). Development was carried out using 0.4 mg/mL O-phenylenediamine dihydrochloride (OPD) in citrate buffer pH 5 with 0.024% hydrogen peroxide (H_2_O_2_) after additional washing with PBS-Tween 0.05%. Finally, the optical density (OD) at 450 nm was measured in a microplate reader (Mindray, Shenzhen, PR China).

### RNA isolation and RNA m^6^A quantification

Total RNA was extracted using TriReagent (Sigma, T9424) according to the manufacturer's protocol. Then, the absolute amount of m^6^A present in total RNA was measured through the ELISA-based EpiQuik m^6^A RNA Methylation Quantification Kit (Epigentek, P-9005) according to the manufacturer's protocol. Quantification was performed via a Nanodrop and Bioanalyzer system (Thermo, Nanodrop 1000), and 200 ng of RNA was added to the assay wells. The m^6^A levels were quantified through measuring absorbance. Calculations were performed based on a standard curve.

### PCR amplification and SMRT sequencing

Using the E.Z.N.A.® Soil DNA Kit (Omega Biotek, Norcross, GA, U.S.), total DNA was extracted from the faecal samples according to the manufacturer's protocols. The V1-V9 region of the bacterial 16S ribosomal RNA gene was amplified via PCR (95 °C for 2 min, followed by 27 cycles at 95 °C for 30 s, 55 °C for 30 s, and 72 °C for 60 s and a final extension at 72 °C for 5 min) using primers 27F 5′-AGRGTTYGATYMTGGCTCAG-3′ and 1492R 5′-RGYTACCTTGTTACGACTT-3′, where the barcode is an eight-base sequence unique to each sample. PCRs were performed in triplicate in a 20 μL mixture containing 4 μL of 5 × FastPfu Buffer, 2 μL of 2.5 mM dNTPs, 0.8 μL of each primer (5 μM), 0.4 μL of FastPfu Polymerase, and 10 ng of template DNA. Amplicons were extracted from 2% agarose gels and purified using an AxyPrep DNA Gel Extraction Kit (Axygen Biosciences, Union City, CA, U.S.) following the manufacturer's instructions. Next, SMRTbell libraries were prepared from the amplified DNA through blunt ligation according to the manufacturer's instructions (Pacific Biosciences). Purified SMRTbell libraries from the Zymo and HMP mock communities were sequenced on dedicated PacBio Sequel II 8 M cells using the Sequencing Kit 2.0 chemistry. Finally, the purified SMRTbell libraries from the pooled and barcoded samples were sequenced on a single PacBio Sequel II cell.

### Statistical analyses

All data are presented as the mean ± standard error (SE). Student's t-tests were used to evaluate the difference between two groups, and a *p value* < 0.05 was considered significant. All analyses were performed using SPSS version 25.0 (IBM, New York, NY, USA). Bar charts display the predominant abundant phyla and species (>1% abundance). The relative abundance was detected between sequencing technologies using a paired Student's t-test. Significant differences in taxa between the CCI and CCI+HBO groups at 10 days post-CCI were compared using linear discriminant analysis effect size (LEfSe), which employs a nonparametric factorial Kruskal-Wallis test with a subsequent unpaired Wilcoxon test. An LDA > 3 and a *p value* < 0.05 were considered significant. Alpha diversity (ACE, Chao, Simpson's, and Shannon indices) was compared between CCI and CCI+HBO samples using a t-test with two dependent means, and the significance level was set at *p* < 0.05. Principal coordinate analysis plots and clustering dendrograms were generated to visualise the beta diversity of the faecal microbiomes in the CCI and CCI+HBO groups.

## Results

### HBO decreases cortical tissue loss while increasing neuronal cell survival and jejunal tissue V/C ratio following CCI

**Figure 1:**
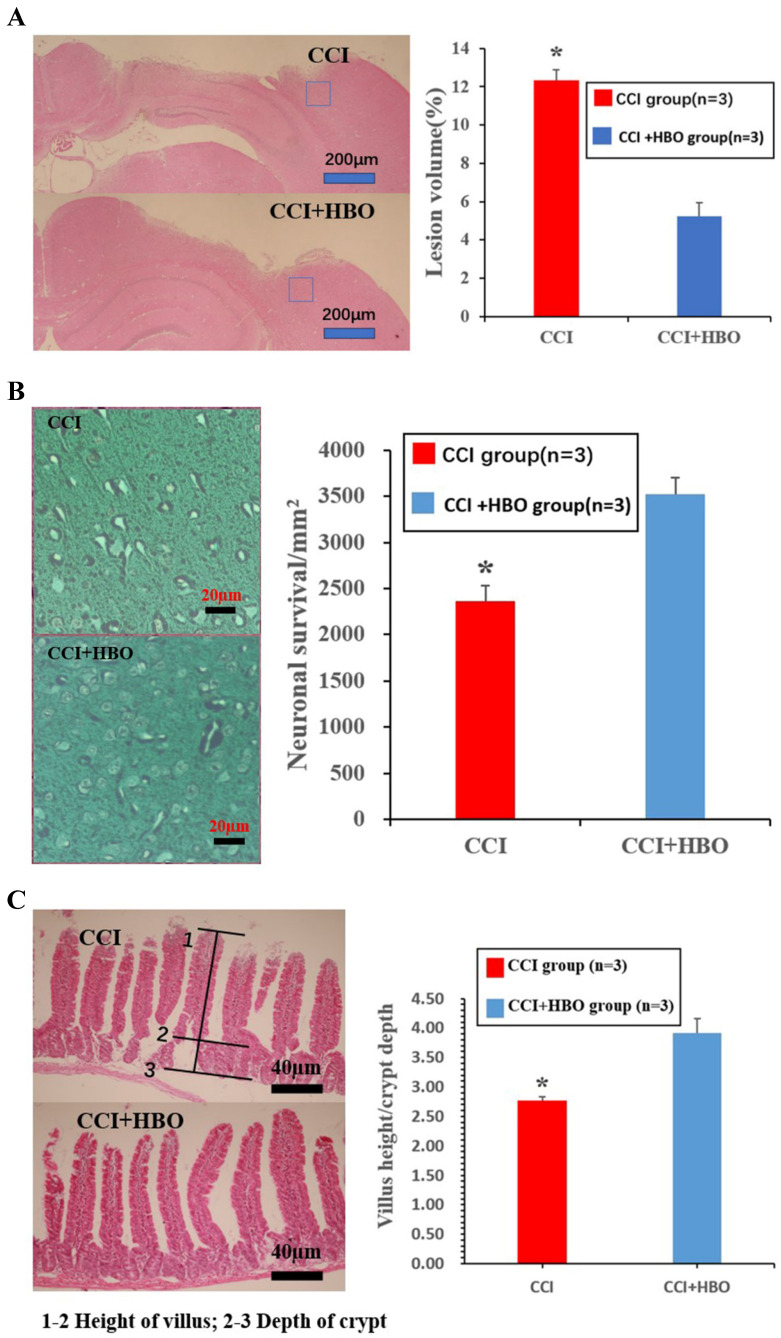
Histological images of HE coronal sections showing the brain tissue loss and neuronal survivals at 10 days post-CCI. (A) The lesion volume increased in CCI group compared to CCI+HBO group (n = 3 mice per group). Scale bars: 200 μm. The lesion volume was quantified (Calculation formula: lesion volume (%) = (healthy side volume-injured side volume) / healthy side volume × 100%). * *p*<0.05. (B) The neuronal survivals decreased in CCI group compared to CCI+HBO group. Scale bars: 20 μm. The number of neuronal survival (5 fields/ section) was quantified. Values are means ±SEs. * *p*<0.05. (C) The villus height decreased and the crypt depth increased in CCI group compared to CCI+HBO group. Scale bars: 40 μm. The V/C ratio (5 fields/ section) was quantified. Values are means ± SEs. * *p*<0.05.

Ten days after CCI, HE staining showed a marked loss of cortical tissue in the CCI group compared with that of the CCI+HBO group (Figure 1A) (*p* <0.05). Meanwhile, the CCI group showed a distinct decrease in the total neuron count in the perilesional zone to trauma compared to that in the CCI+HBO group (Figure 1B). In addition, villus height decreased, while crypt depth increased in the jejunal tissue, and the V/C ratio of the CCI group decreased markedly compared with that of the CCI+HBO group (Figure 1C).

### HBO decreases inflammation and oedema protein level of jejunal tissue post-CCI

**Figure 2:**
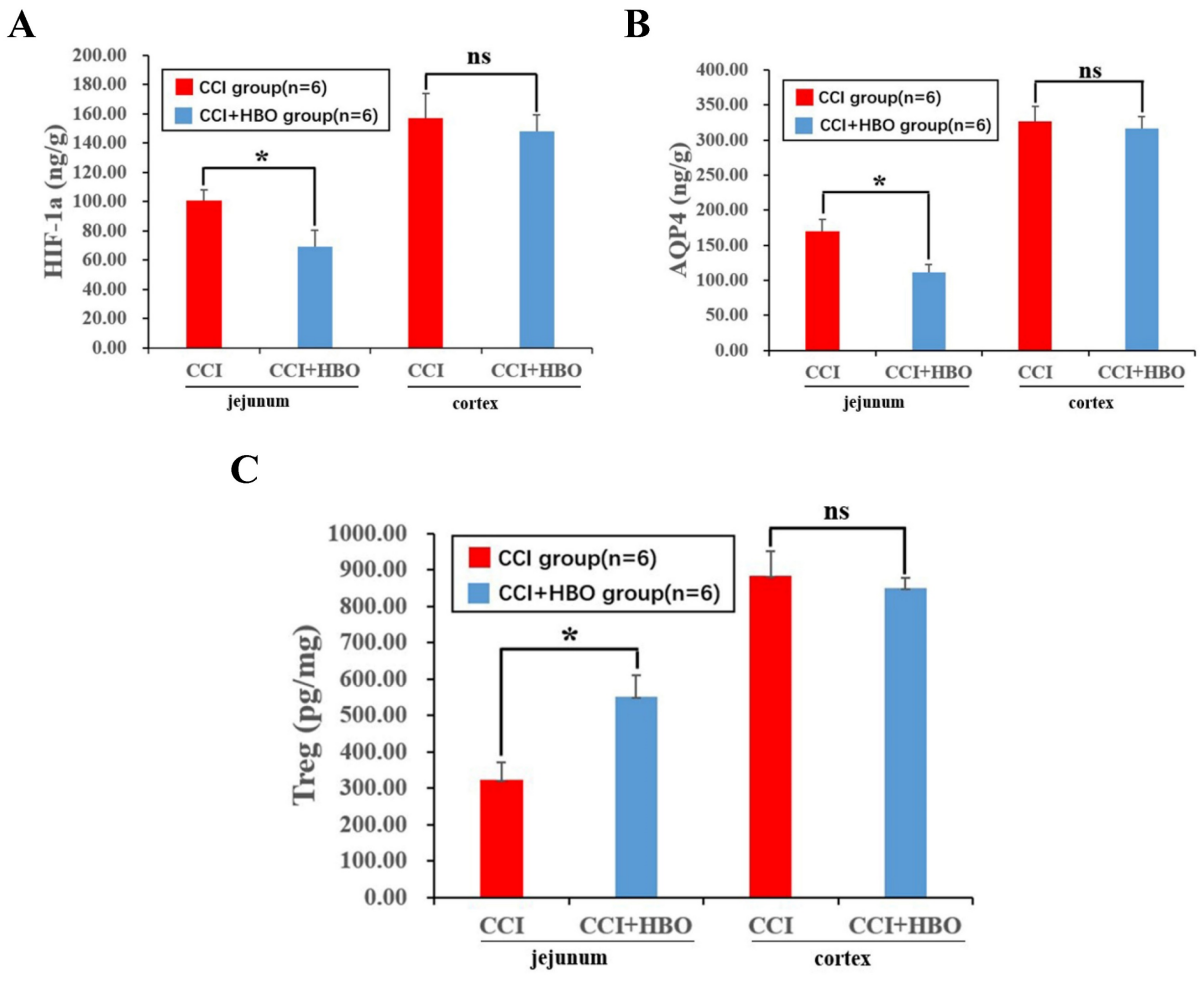
ELISA revealed that HBO significantly affected the inflammation and edema protein level in jejunal tissue at 10 days post-CCI. (A) Compared to CCI group, the inflammation protein HIF-1a level of CCI+HBO group was decreased markedly in jejunal tissue while there was no notable difference in injured cortical tissue. (B) Compared to CCI group, the edema protein AQP4 level of CCI+HBO group was decreased significantly in jejunal tissue while there was no marked difference in injured cortical tissue. (C) Compared to CCI group, the Treg level of CCI+HBO group was increased s markedly in jejunal tissue while there was no significant difference in injured cortical tissue. Values are means ± SEs. *P < 0.05, ns = not significant.

Ten days after CCI, ELISA analysis revealed a notable decrease in inflammatory proteins, including hypoxia-inducible factor 1-alpha (HIF-1a) and the oedema protein aquaporin 4 (AQP4), while regulatory T cells (Treg) showed a significant increase in the CCI+HBO group compared to that in the CCI group in jejunal tissue. Intriguingly, there was no marked difference in injured cortical tissue between the CCI+HBO and CCI groups (Figure 2A-C).

### HBO increases microbiome abundance, and changes microbiome structure and microbiome colonization after CCI

**Figure 3:**
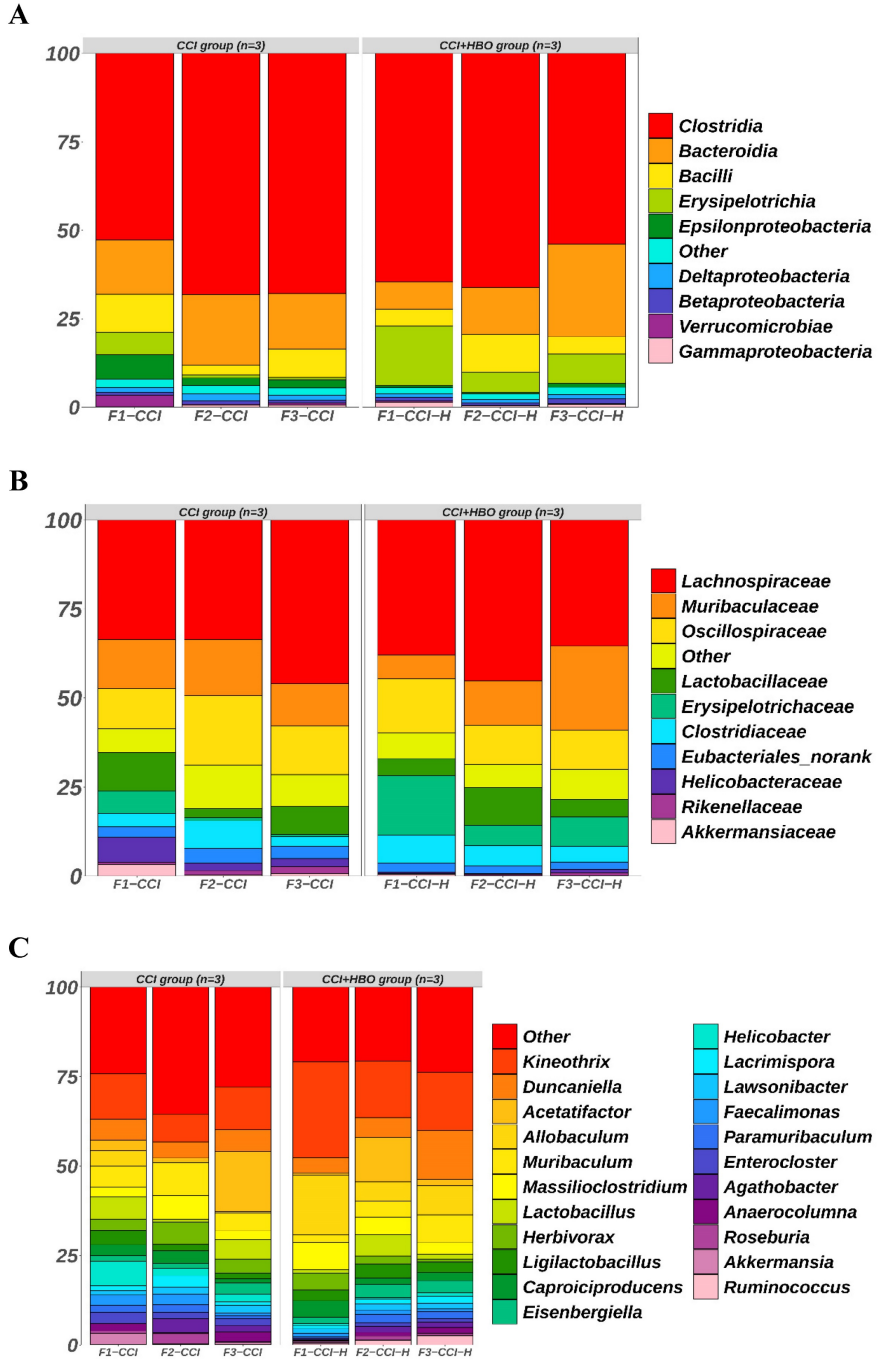

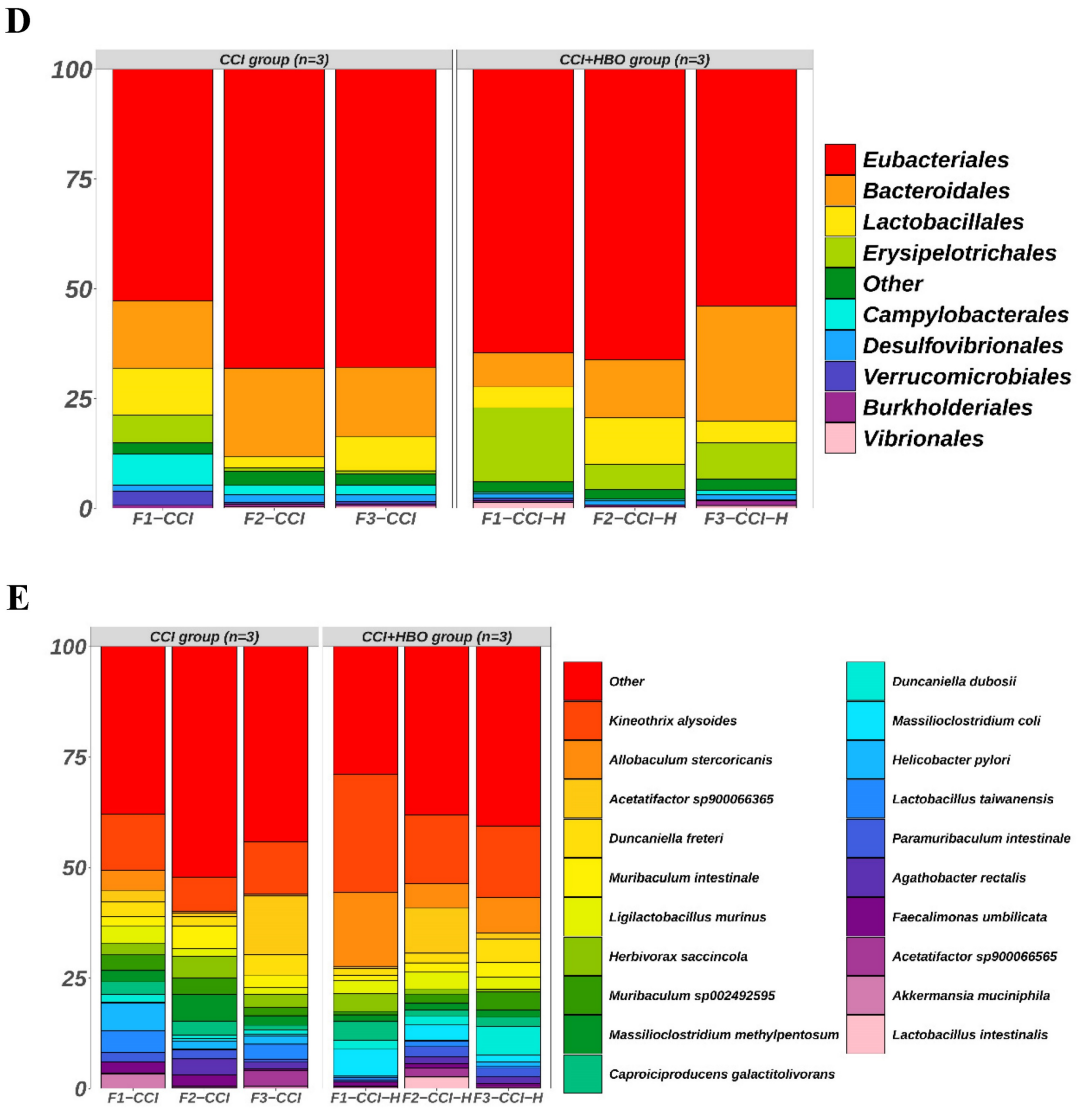
The bar plots reveal the relative abundance of microbiomes in fecal samples of CCI+HBO mice and CCI mice at 10 days after CCI (A = class, B = family, C = genus, D = order, and E = species), which were detected by high throughput sequencing on Internal transcribed spacer 2 (ITS2) of ribosomal DNA and analyzed by QIIME2 pipeline.

The raw data from Illumina sequencing were processed using the QIIME2 pipeline, and their relative abundance was calculated and grouped according to mouse origin (Figure 3). The results indicated different profiles of communities in the faecal microbiomes of CCI+HBO and CCI mice 10 days after CCI. At the class level, the most abundant microbiomes in the CCI+HBO group were Clostridia (61.5%), Bacteroidia (15.8%), Erysipelotrichia (10.3%), and Bacilli (6.8%), while the CCI group microbiomes were dominated by Clostridia (62.9%), Bacteroidia (17.0%), Bacilli (7.2%), and Erysipelotrichia (2.6%). At the family level, the most abundant microbiomes in the CCI+HBO group were Lachnospiraceae (39.5%), Muribaculaceae (14.2%), and Oscillospiraceae (12.4%), while the CCI group microbiomes were dominated by Lachnospiraceae (37.7%), Oscillospiraceae (14.8%), and Muribaculaceae (13.7%). At the genus level, the most abundant microbiomes in the CCI+HBO group were *Kineothrix* (19.5%), *Allobaculum* (10.1%), *Duncaniella* (7.9%), and *Acetatifactor* (4.9%), while the CCI group microbiomes were dominated by *Kineothrix* (10.9%), *Acetatifactor* (6.9%), *Duncaniella* (5.4%), and *Allobaculum* (1.7%). At the order level, the most abundant microbiomes in the CCI+HBO group were Eubacteriales (61.5%), Bacteroidales (15.8%), Erysipelotrichales (10.3%), and Lactobacillales (6.8%), while the CCI group were dominated by Eubacteriales (62.9%), Bacteroidales (17.0%), Lactobacillales (7.1%), and Erysipelotrichales 2.6%). At the species level, the most abundant microbiomes in the CCI+HBO group were *Kineothrix alysoides* (19.5%), *Allobaculum stercoricanis* (10.1%), *Acetatifactor* sp900066365 (4.0%), and *Duncaniella freteri* (3.0%), while the CCI group microbiomes were dominated by *Kineothrix alysoides* (10.7%), *Acetatifactor* sp900066365 (5.5%), *Duncaniella freteri* (3.4%), and *Allobaculum stercoricanis* (1.7%).

**Figure 4:**
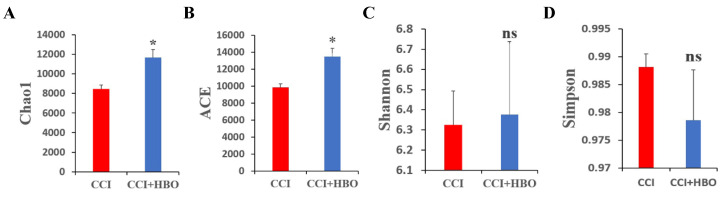
The comparison of alpha diversity of microbiome in in fecal samples of CCI+HBO mice and CCI mice at 10 days following CCI calculated by 4 different indices: A = Chao1 index, B = ACE index, C = Shannon's index, and D = Simpson's index. The bars show average diversity with standard error of each group and statistically significant difference (*P < 0.05, ns = not significant).

For alpha diversity, the Chao1 and ACE indices indicated the abundance of the microbiome, whereas Shannon's and Simpson's indices demonstrated the diversity of the microbiome. The results showed that the Chao1 and ACE indices of faecal microbiomes in CCI+HBO mice were significantly higher than those in CCI mice at 10 days post-CCI (*p* < 0.05) (Figure 4A-B). However, Shannon's and Simpson's indices of faecal microbiomes in CCI+HBO and CCI mice were similar (Figure 4C-D).

**Figure 5:**
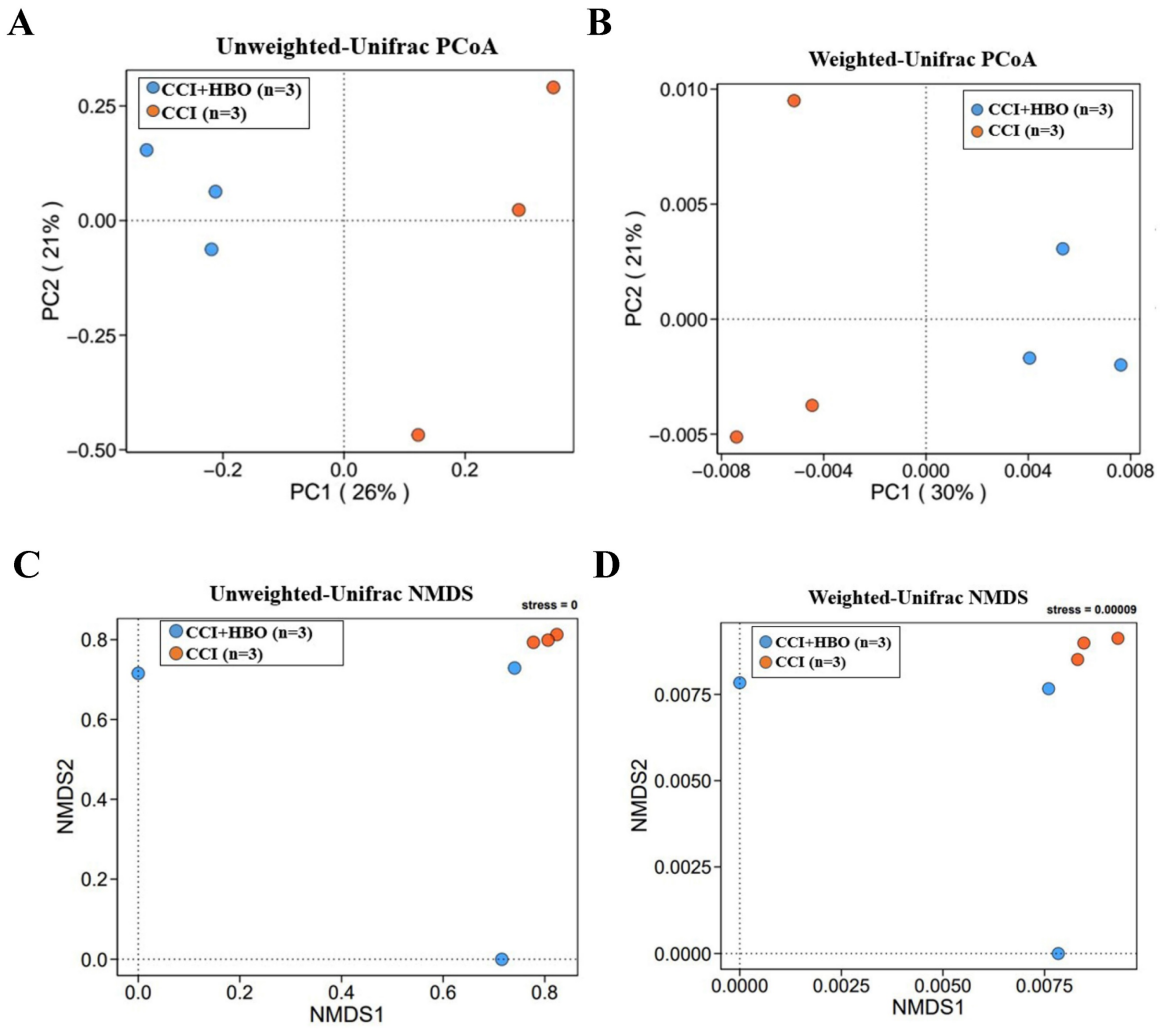
The beta diversity of faecal microbiome between samples was showed by principle coordinate analysis (PCoA) and non-metric multidimensional scaling (NMDS) plots of A+C unweighted and B+D weighted Unifrac distance illustrating marked difference of faecal microbiome profiles between CCI+HBO and CCI groups (tested by Permanova analysis with *P* < 0.05).

The beta diversity exhibiting the community distance between samples was evaluated by unweighted and weighted UniFrac distances (Figure 5A-D), which indicated a marked difference (*p* < 0.05) in the microbiome profiles between the CCI+HBO and CCI groups at 10 days after CCI.

**Figure 6:**
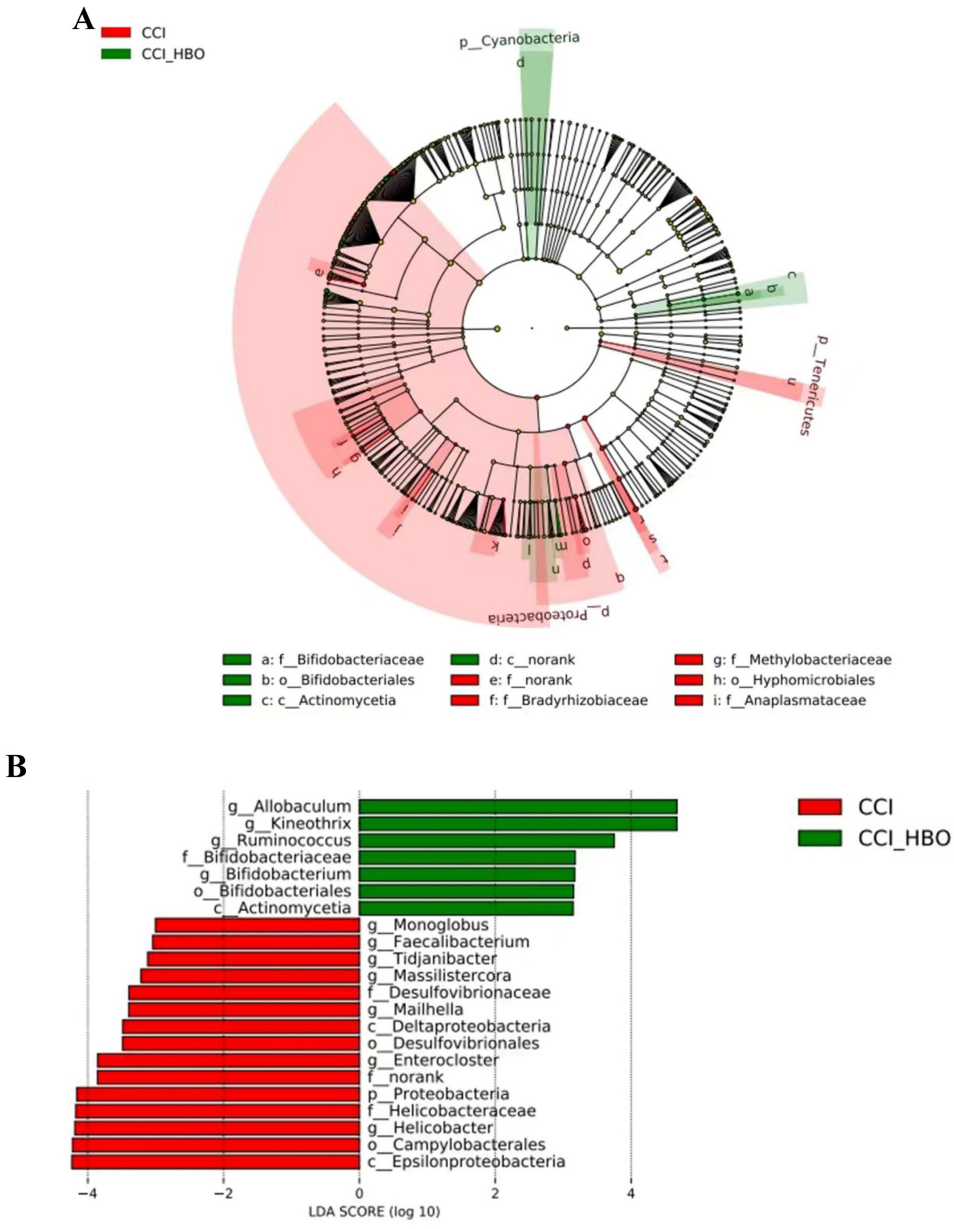
LEfSe analysis of fecal microbiome of mice at 10 days after CCI shows the significantly differential taxa between CCI+HBO and CCI mice. A Cladogram (2-fold, P < 0.05). B LEfSe analysis for differential abundant taxa detected between CCI+HBO and CCI groups. Threshold parameters were set as P =0.05 for the Mann-Whitney U test and multiclass analysis=all against all. Linear discriminant analysis (LDA) score >3.0. (Green color labels demonstrate the enriched fungal taxa in CCI+HBO while the red labels indicate the taxa enriched in CCI group.

Differential taxa between CCI+HBO and CCI mice were analysed using the LefSe method. The results in Figure 6A illustrate the enriched microbiome taxa in each group that had a >2-fold change and* P* < 0.05 (Kruskal‒Wallis test). The faecal microbiome of the CCI+HBO mice diverged significantly from that of the CCI mice 10 days after CCI (Figure 6B), indicating diverse microbiome colonisation.

### HBO increases m^6^A level in brain tissue following CCI

**Figure 7:**
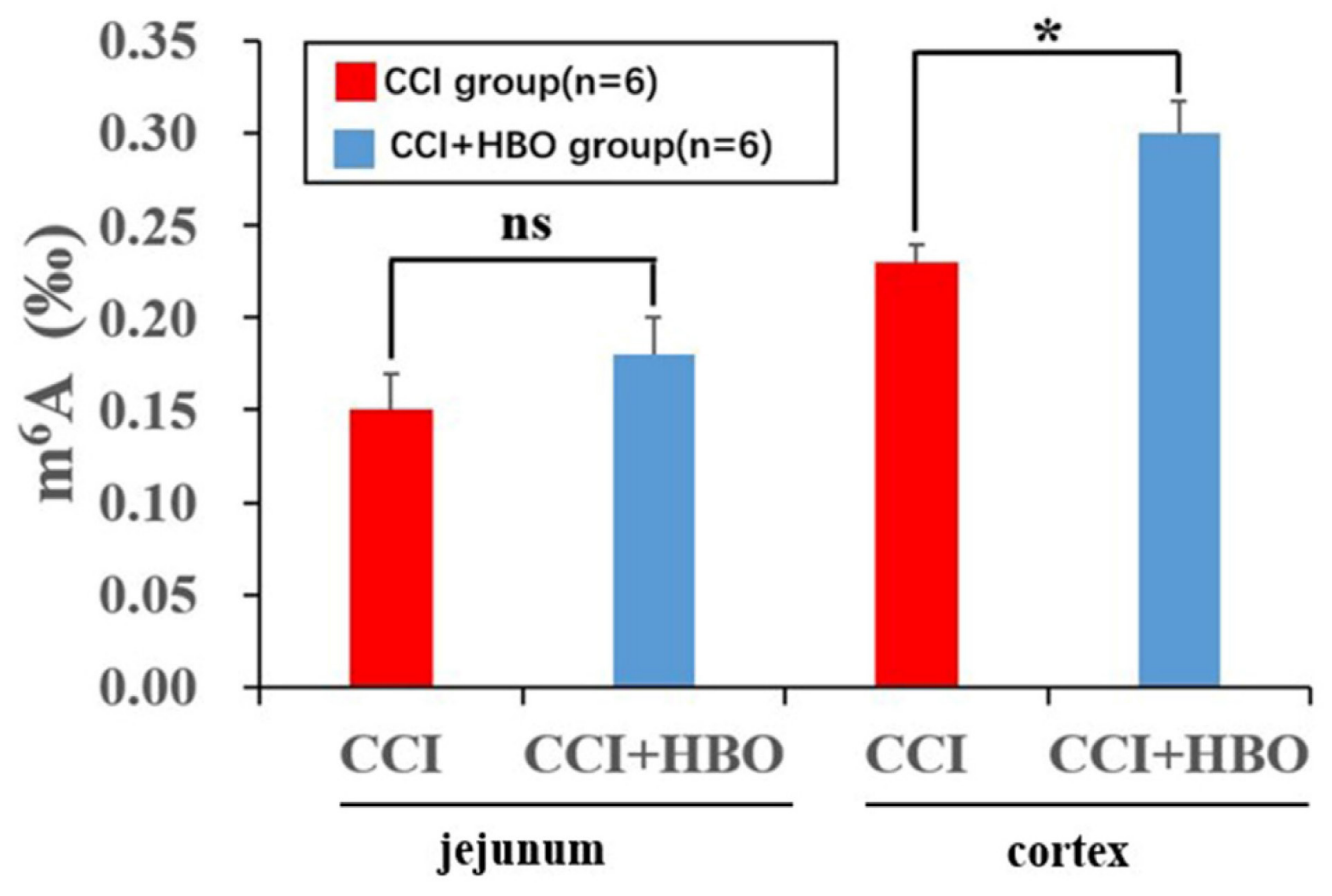
ELISA showed that HBO notably affected the m^6^A level in injured cortical tissue while there was no marked difference in jejunal tissue at 10 days after CCI.Values are means ± SEs. *P < 0.05, ns = not significant.

ELISA analysis revealed that the total m^6^A level increased markedly in the CCI+HBO group compared to that in the CCI group in injured cortical tissue 10 days after CCI. However, there was no notable difference between the CCI+HBO and CCI groups in jejunal tissue (Figure 7).

## Discussion

Several studies have focused on the role of HBO in the treatment of various neurological diseases such as stroke^18^-^20^, intracerebral haemorrhage^21^, glioma^22^,^23^, Alzheimer's disease^24^,^25^, Parkinson's disease^26^,^27^, cerebral palsy^28^,^29^, and TBI^30^-^33^. In addition, numerous studies have confirmed that HBO therapy can improve intestinal dysfunction after spinal cord injury^9^,^34^ and chronic stress^10^. However, few studies have focused on the role of HBO in intestinal dysfunction following TBI. We observed that HBO therapy reduced histopathological lesions and decreased the levels of inflammation and oedema proteins in the intestinal tissues of mice 10 days after TBI. It also improved microbiome abundance and probiotic colonisation in mice post-CCI. We found that HBO increased the level of m^6^A in injured cortical tissue, suggesting that m^6^A may be involved in the regulation of TBI recovery after HBO treatment.

After TBI, TBI-induced neuroinflammation affects gut function via BGA^35^. Our results showed that HBO attenuated structural lesions in both brain and intestinal tissues following TBI. At the protein level, hypoxic conditions induce HIF-1a expression, which regulates the releases of inflammatory cytokines^36^. Tregs are a special family of inhibitory CD4+T cells that act as key negative regulators of inflammation in various biological environments. Tregs show a strongly enhanced inhibitory function when exposed to inflammation^37^. HBO therapy boosts Treg expression while reducing HIF-1α expression in mice with antigen and collagen-induced arthritis^38^. In addition, AQP4 expression increases in TBI^39^ and colonic inflammation^40^, and AQP4-knockout attenuates experimental colitis in mice^41^. Our results revealed that HBO blocked intestinal inflammation and oedema in mice 10 d after TBI. However, the expression of inflammatory and oedema proteins in the brain tissue was not significantly different between the CCI+HBO and CCI groups at 10 days post-TBI. We speculated that 10 days after CCI, although inflammation and oedema reactions in brain tissue had subsided without HBO treatment^42^-^44^, they persisted in intestinal tissue.

The gut microbiome is a rich and complex ecosystem composed of viruses, archaea, protists, bacteria, fungi, and (occasionally) helminths^45^. Gut bacteria are critical for microbiome-BGA^46^, and the fungi equilibrium is crucial for microbiome stability^47^. Microbiome interactions may be involved in the microbiome-BGA communication through immune- and nonimmune-modulated crosstalk systems^48^. Substantial evidence has revealed that TBI can affect the gut microbiome by disrupting BGA^49^, and host m^6^A modifications can induce gut inflammatory responses to alter the gut microbiome^2^,^50^. M^6^A could participate in the interaction between the host and microbiome, along with noncoding RNAs, histone modifications, and chromatin remodelling^51^.

In addition, HBO can remodel the gut microbiome and regulate host metabolism to improve depression-like behaviour in a chronic stress mouse model^52^. Thus, we observed alterations in the faecal microbiome and m^6^A levels following HBO treatment for TBI. This study results indicated that the diversity of the microbiome was markedly altered in CCI+HBO and CCI mice at 10 days after CCI. In alpha diversity, the Chao1 and ACE indices of the CCI+HBO mice were significantly higher than those of the CCI mice, whereas Shannon's and Simpson's indices were similar; hence, HBO enhanced the abundance of the microbiome but not its diversity of the microbiome post-CCI. Beta diversity analysis revealed the specific microbiome structure identified in the group samples. The unweighted and weighted UniFrac distances showed that the faecal microbiomes of CCI+HBO and CCI mice had various community structures. The distinct characteristics of the microbiome can be detected from the class to the species level. The results of this study demonstrated that the microbiome composition varied between the CCI+HBO and CCI groups.* Allobaculum, Kineothrix, Ruminococcus, Bifidobacterium,* and *Actinomycetia* showed significant enrichment in CCI+HBO mice (Figure 6B). *Allobaculum* was one of the intestinal genera that are most sensitive to changes in host diet and was strongly inversely correlated with circulating leptin and expression of several genes that correlated with energy expenditure and inflammation^53^. *Kineothrix* produces butyrate, a metabolite that serves as energy source of enterocytes and has notable anti-inflammatory and immunomodulatory properties^54^. *Ruminococcus* serve to degrade and convert complex polysaccharides into a variety of nutrients for their hosts^55^. *Bifidobacterium* has long been regarded as a probiotic that modulates the microbial structure to improve gut health^56^. *Actinomycetia* play major roles in soil and plant health 57. In addition, *Allobaculum*^58^*, Kineothrix*^59^*,* and *Ruminococcus*^55^ are strictly-anaerobic, and Bifidobacterium is obligate anaerobic^60^, while Actinomycetia is aerobic^57^. The bacterial species dominating the microbiota in the gut are strict anaerobes^61^. However, gut inflammation can induce dysbiosis, which is characterized by significantly decreased obligate anaerobic bacteria and markedly increased facultative anaerobic bacteria^62^. Dysbiosis is the result of the oxidative nature of the host inflammatory response^63^. In addition, the composition of the gut microbiota is regulated by the oxygen^64^. Yong Li *et al.* found in a study on hyperbaric oxygen treatment for Crohn's disease that the relative abundance of *Bifidobacterium* increased after HBO treatment while the anaerobic or aerobic nature of microbes did not represent the trend of the population in their host after HBO treatment^65^. HBO can modulate mitochondrial redox, maintain mitochondrial integrity, catalyze transcription factors, alleviate oxidative stress, and facilitate anti-inflammatory effects following TBI^66^, which may improve post-TBI dysbiosis. The interactions between the gut microbiota and host during the process of HBO treatment after TBI should be further clarified.

Moreover, m^6^A RNA modification is an important mechanism in the interaction between the gut microbiota and the host^67^. *Bifidobacterium* and *Lactobacillus* species can synthesise folate to enhance gut m^6^A levels, which promotes normal gut development^56^. In this study, the results showed that the m^6^A level increased notably in the injured cortical tissue of the CCI+HBO group compared to that in the CCI group, but there was no significant difference in jejunal tissue between the CCI+HBO and CCI groups at 10 days after CCI (Figure 7). Therefore, we speculated that HBO therapy might alleviate TBI-induced intestinal dysfunction via m^6^A-mediated BGA signalling pathway.

## Conclusion

Our study provides new insights into HBO treatment for TBI-induced intestinal dysfunction. Further studies are necessary to explore the mechanisms of m^6^A involved in HBO curing intestinal dysfunction following TBI. This study had several limitations. Firstly, the expression of inflammation and oedema proteins between the CCI+HBO and CCI groups in the brain tissue was not markedly different at 10 days following TBI; therefore, a suitable sampling time point is required. Secondly, we only described the alteration of total m^6^A levels between the CCI+HBO and CCI groups after TBI; changes in the specific gene levels of m^6^A between the CCI+HBO and CCI groups remain to be explored in future studies. Thirdly, our study only included male mice, and data from female mice should be collected. In summary, HBO therapy could alleviate TBI-induced intestinal dysfunction, and m^6^A might play a role in the regulatory mechanisms of HBO treatment for intestinal dysfunction following TBI.

## Supplementary Material

Supplementary figure.
