# Supplementary material for: Hyperbaric oxygen therapy alleviates intestinal dysfunction following traumatic brain injury via m6A regulation
Source: Int J Med Sci. 2024 Aug 19;21(12):2272–84. doi: 10.7150/ijms.97682 (PMC11413893; doi:10.7150/ijms.97682)
Supplement: Supplementary file 1 — Supplementary figure. [file ijmsv21p2272s1.pdf]

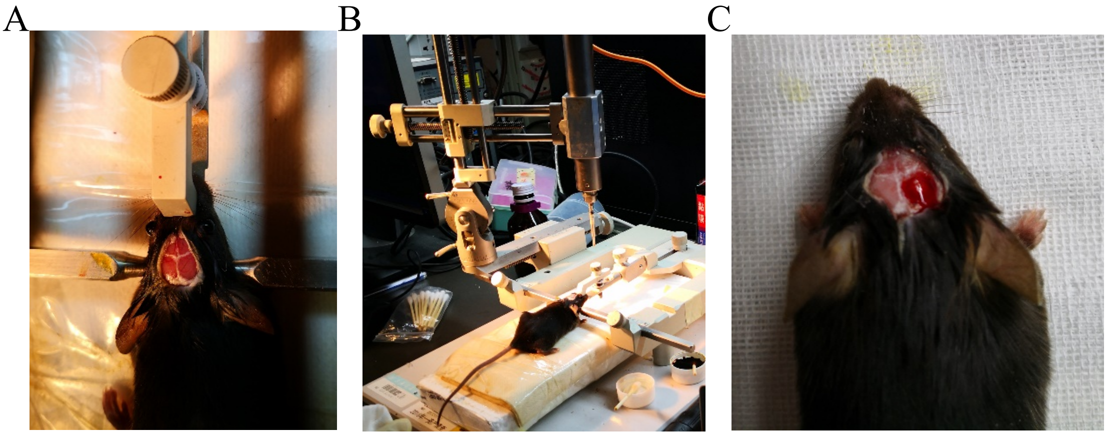

Supplemental figure 1. CCI Procedure. (A) The head of the mouse was fixed in a stereotaxic frame, and a 10-mm-length incision was performed along the midline. (B) A 4-mm-diameter craniotomy was performed at 2.0 mm posterior to the bregma and 2.0 mm lateral to the midline over the right hemisphere and a 3.0-mm rounded metal tip attached to the Pin-Point CCI device was angled vertically towards the brain surface. (C) A severe injury was performed successfully.
